# Madderized Bone for Bone Grafts

**Published:** 1918-10

**Authors:** 


**Madderized Bone as a Material for Bone Grafts.** By Madge DeG. Thurlow and C. C. Macklin, from the Dept. of Anatomy, Johns Hopkins University. Abs. frcm the *Annal of Surgery,* April 1918.

The animal should be fed madder from birth until the time the bone is removed.

Rats eat the dye stuff readily and therefore were used in this series of experiments. Only transplantation into the flat bones of the skull was done. Two animals were used: one being killed 55 days after operation, the other 5 months after. In both cases the inserts show very clearly, as they have kept their color and have been welded into place by unstained callus.

It is suggested that it would be possible to observe the rapidity of displacement of a graft in a series of cases by putting an unstained graft into an unstained animal and putting the animal on a madder diet until killed, with the result that all new bone will be colored.

